# Sleep duration and prevalence of coronary artery disease among adults in Chongqing, China

**DOI:** 10.3389/fepid.2026.1671078

**Published:** 2026-03-02

**Authors:** Jie He

**Affiliations:** Beijing Anzhen Nanchong Hospital, Capital Medical University & Nanchong Central Hospital, Nanchong, China

**Keywords:** China, coronary artery disease, epidemiology, public health, sleep duration

## Abstract

**Objective:**

This study aimed to explore the association between sleep duration and prevalence ofcoronary artery diseases (CAD) among adults in Chongqing, China, and discuss implications for clinical practice and public health policy.

**Methods:**

Baseline variables were collected from 2,320 adults who participated in community medical examinations in Chongqing, China, between August 2018 and October 2020. Sleep duration was self-reported and categorized into short (<6 h/day), normal (6–8 h/day), and long (>8 h/day). Multivariate logistic regression was used to examine associations between sleep duration and CAD, adjusting for demographic and clinical confounders.

**Results:**

Short sleep (<6 h/day; OR = 1.595, 95% CI = 1.230–2.067) and long sleep (>8 h/day; OR = 2.284, 95% CI = 1.456–3.583) were significantly associated with increased odds of CAD compared to normal sleep duration (6–8 h/day), even after adjusting for confounders. Long sleep duration demonstrated a notably stronger association with CAD risk.

**Conclusion:**

Both short and long sleep durations are significant risk factors for coronary artery diseases, with longer sleep duration showing a stronger association. Public health initiatives and clinical practices should integrate sleep duration assessments to identify at-risk populations and implement targeted interventions.

## 1 Introduction

Sleep plays a critical role in maintaining well-being, with individuals spending roughly one-third of their lives asleep. However, in today's fast-paced society, extended work hours and increased job demands have led to reduced sleep for many, contributing to a rise in sleep disorders. Sleep deprivation (SD) has emerged as a significant health concern in modern communities (1). Poor sleep is linked to negative health outcomes (2), such as type 2 diabetes (3), hypertension (4), and various metabolic disorders (5). A growing body of evidence suggests a close link between sleep duration and cardiovascular diseases, though findings on short vs. long sleep duration are inconsistent. Some research indicates that both very short and very long sleep durations elevate the risk of cardiovascular diseases (6–13). Specifically, sleep durations outside the 6–8 h range are strongly associated with adverse cardiovascular outcomes, including myocardial infarction, heart failure, and cardiovascular death (12, 13). In a recent large cohort study conducted in Korea, both short and long sleep were shown to increase the risk of major cardiovascular events, emphasizing the importance of maintaining optimal sleep patterns for cardiovascular health (8). This study, conducted in Chongqing, China, aims to address the gap in regional data by examining the association between sleep duration and CAD among Chinese adults.

Conversely, other studies offer conflicting findings, with some showing that only short (14, 15) or only long (16, 17) sleep duration correlates with a higher incidence of cardiovascular diseases. This study aims to determine whether self-reported sleep duration is associated with cardiovascular diseases among adults in Chongqing, China.

Although meta-analyses have explored sleep duration and CAD risk, regional differences remain understudied, especially within the Chinese population. Our study uniquely addresses this gap by examining a community-based sample in Chongqing, China. This region-specific study contributes novel evidence that is essential for tailored interventions and clinical recommendations within local contexts.

## 2 Methods

**Figure 1:**
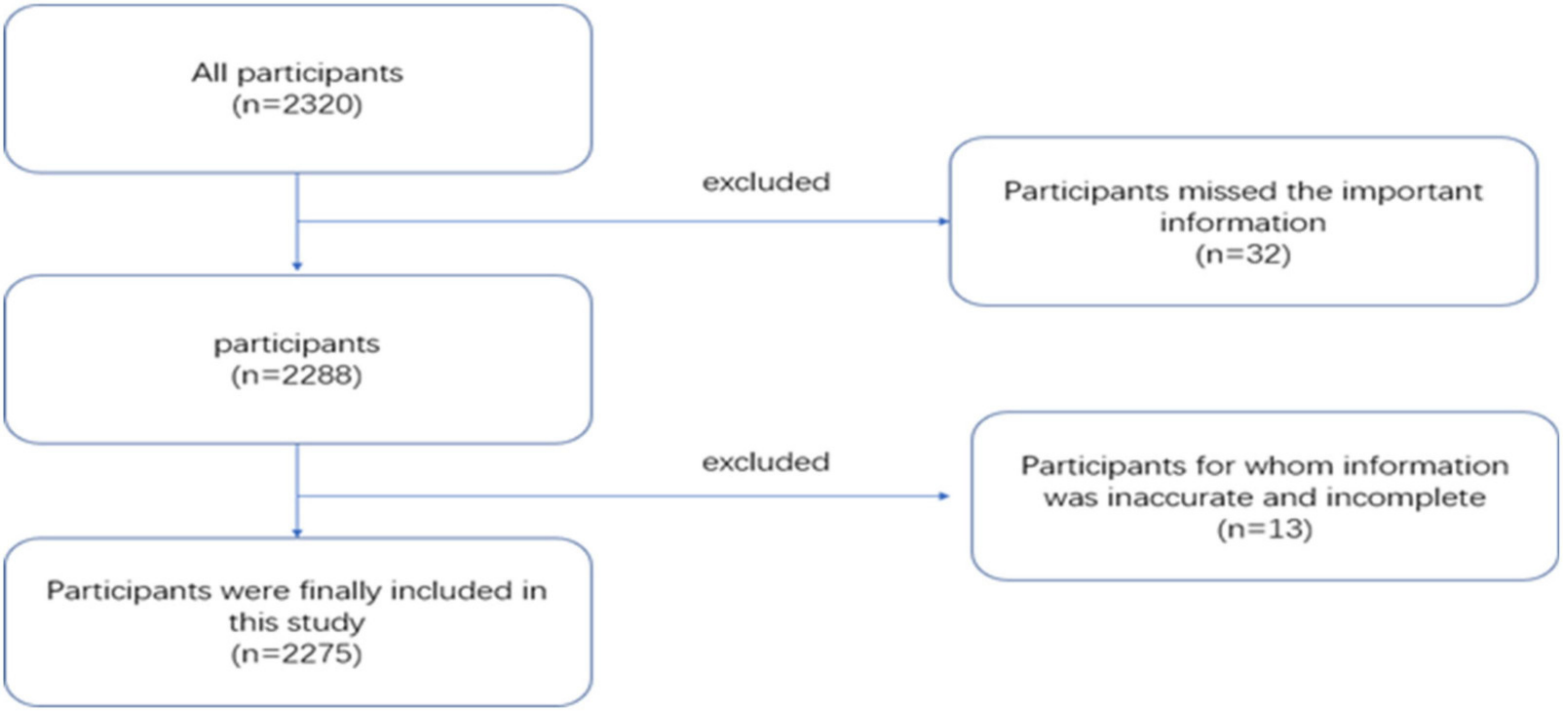
Flowchart of study participants.

Study Population We conducted a cross-sectional study including 2,320 adults (aged 31–96) who participated in community medical examinations in Chongqing from August 2018 to October 2020. Participants with missing or inaccurate data (*n* = 45) were excluded, resulting in 2,275 participants (Figure 1).

Definition of coronary artery disease (CAD) was specifically defined as coronary artery disease (CAD), including myocardial infarction, acute coronary syndrome, chronic stable angina, or coronary artery stenosis greater than 50%.

Sleep Duration Assessment Sleep duration was self-reported, categorized into short (<6 h/day), normal (6–8 h/day), and long (>8 h/day) sleep durations. Normal sleep duration (6–8 h/day) was used as a reference.

Covariates included age, sex, BMI, smoking, drinking, hyperlipidemia, diabetes, hypertension, physical activity, dietary habits, and suspected sleep apnea. However, due to limitations in available data, physical activity, dietary habits, and sleep apnea were not included in the statistical models. These factors are known to influence cardiovascular health and may act as confounders. Future studies should consider collecting data on these factors for more comprehensive analyses. Clinical measurements (e.g., blood pressure, blood glucose levels) were used to validate self-reported conditions.

## 3 Statistical analysis

Data were analyzed using SPSS (v26.0). Logistic regression models adjusted for covariates were applied to assess associations between sleep duration and CAD risk. Sensitivity analyses were conducted stratified by sex, age, and BMI categories to ensure robustness. For handling missing data, we employed the **Complete Case Analysis (CCA)** approach, which includes only participants with no missing data in the analysis. This method is straightforward and commonly used; however, it can lead to reduced sample size if there is substantial missing data, potentially affecting the statistical power and generalizability of the results.

## 4 Results

### 4.1 Baseline characteristics of all participants

The baseline characteristics of all participants are shown in Table 1. A total of 2,275 participants aged 31–96 years were included in this study. The overall prevalence of coronary artery disease (CAD) was 49.4%, which appears high for a general population and may indicate a selection bias. The study population may not be representative of the general community, and the results should be interpreted with caution. This discrepancy suggests that the sample may have been drawn from a clinical or high-risk setting. Further clarification is provided in the “Study Population” section below. Participants with coronary artery disease (CAD) were predominantly female, older, and had a higher BMI and hypertension. Analysis of the baseline characteristics of all participants showed that age, BMI, drinking, sleep duration, hyperlipidemia, and hypertension all differed significantly between the two groups (*P* < 0.05). However, there were no significant differences with regard to sex, smoking, and diabetes, (*P* > 0.05).

**Table 1:** Baseline characteristics of study participants by CAD status.

Variable	Cardiovascular disease		*P*
	Yes	No	
Sex (%)			
Male	448 (39.9)	482 (41.9)	0.35
Female	676 (60.1)	669 (58.1)	
Age	74 ± 8	64 ± 11	<0.01
BMI	25 ± 4	25 ± 3	<0.01
Smoking (%)			
Yes	201 (17.9)	201 (17.5)	0.83
No	1,425 (13.9)	1,464 (15.0)	
Drinking (%)			
Yes	122 (10.9)	166 (14.4)	<0.01
No	1,002 (89.1)	985 (85.6)	
Sleep duration (%)			
6–8 h	795 (70.7)	997 (86.6)	<0.01
<6 h	252 (22.4)	120 (10.4)	
>8 h	77 (6.9)	34 (3.0)	
Diabetes (%)			
Yes	255 (22.7)	251 (21.8)	<0.62
No	869 (77.3)	900 (78.2)	
Hyperlipidemia (%)			
Yes	394 (35.1)	218 (18.9)	<0.01
No	730 (64.9)	933 (81.1)	
Hypertension (%)			
Yes	812 (41.4)	476 (41.4)	<0.01
No	312 (27.8)	675 (58.6)	

*P* < 0.05 was considered significant. BMI, body mass index.

### 4.2 Unadjusted and adjusted analysis of the association between sleep duration and CAD

There were significant differences among the three sleep-duration groups in terms of coronary artery disease (CAD) (χ^2^ = 86.0, *P* < 0.01) (Table 2). Univariate analysis revealed that participants with short (<6 h/day) and long (>8 h/day) sleep durations were significantly associated with greater risks of coronary artery disease (CAD) compared to the participants who reported normal (6–8 h/day) sleep duration. The odds of having coronary artery disease (CAD) were significantly higher by 184.0% in participants with a long (>8 h/day) sleep duration compared to those with a normal (6–8 h/day) sleep duration (OR = 2.840, 95% CI = 1.877–4.298) >8 h/day, and The odds of having coronary artery disease (CAD) were significantly higher by 163.4% in participants with a short (<6 h/day) sleep duration compared to those with a normal (6–8 h/day) sleep duration (OR = 2.634, 95% CI = 2.079–3.336) <6 h/day.

**Table 2:** The unadjusted analysis for the prevalence of CAD by sleep duration.

	Total	CAD (%)		x^2^ test		Logistic regression		
		No	Yes	x^2^	*P*	OR	*P*	95% CI
6–8 h	1,792	795 (70.7)	997 (86.6)	86.0	<0.01	Ref		
<6 h	372	252 (22.4)	120 (10.4)			2.634	<0.01	2.079–3.336
>8 h	111	77 (6.9)	34 (3.0)			2.840	<0.01	1.877–4.298

**Figure 2:**
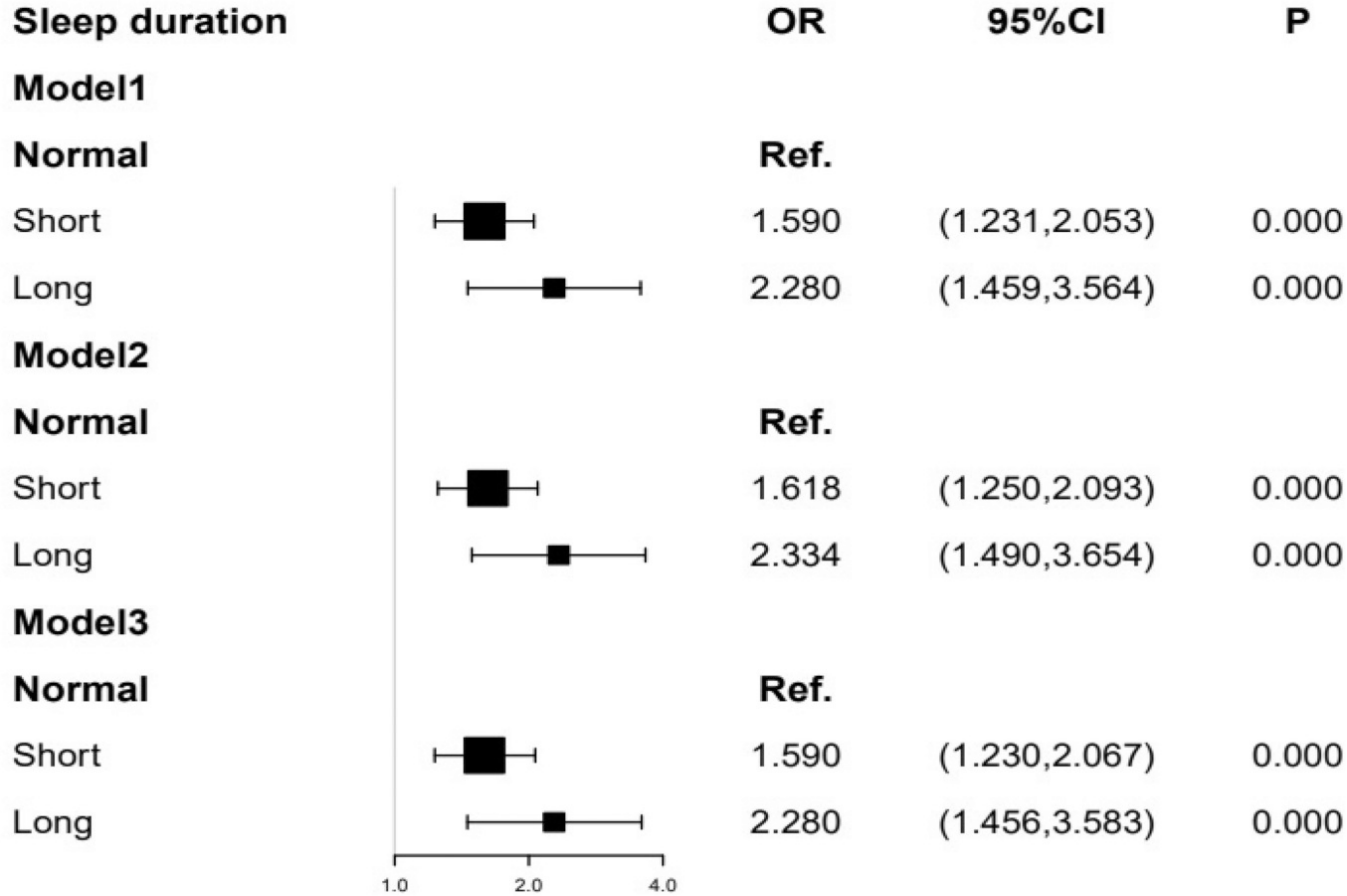
To evaluate the association between sleep duration and CAD by four multivariate logistic regression models. Model 1 included age, BMI, and smoking. Model 2 included model 1 plus hyperlipidemia. Model 3 included model 2 plus hypertension. The odds ratios (OR) and 95% confidence interval (95% CI) were presented in Figure 2.

Figure 2 represented that the risk of CAD was significantly increased by 128.0% in participants with a long (>8 h/day) sleep duration compared with those with a normal (6–8 h/day) sleep duration after adjusting for age, BMI, smoking (OR = 2.280, *P* < 0.01, 95% CI = 1.459–3.564), and the increase of 59.0% in participants with a short (<6 h/day) sleep duration (OR = 1.590, *P* < 0.01, 95% CI = 1.231–2.053). Similar results were obtained after adding Hyperlipidemia (model 2), and hypertension (model 3) as confounding factors. After fully adjusting for confounding factors (model 3), the odds of having coronary artery disease (CAD) were significantly increased by 128.4% in participants with a long (>8 h/day) sleep duration compared with those with a normal (6–8 h/day)sleep duration (OR = 2.284, *P* < 0.01, 95% CI = 1.456–3.583), and the increase of 59.5% in participants with a short (<6 h/day) sleep duration (OR = 1.595, *P* < 0.01, 95% CI = 1.230–2.067).

## 5 Discussion

This study confirms the significant association between both short and long sleep durations and increased risk of coronary artery disease (CAD). Our findings are also in agreement with a recent meta-analysis, which suggested that long sleep duration may have a stronger association with CVD than short sleep duration (18).Consistent with global literature, our findings suggest a stronger association between prolonged sleep duration and CAD risk. Recent meta-analyses have consistently demonstrated that both insufficient sleep (<6 h) and excessive sleep (>8 h) are associated with increased cardiovascular risk, including myocardial infarction, heart failure, and mortality (13, 19). A U-shaped relationship between sleep duration and cardiovascular events has been observed in numerous studies, further supporting our findings (12, 20).

The potential mechanisms underlying these associations include sympathetic overactivity, insulin resistance, inflammation, metabolic syndrome, decreased physical activity, and underlying conditions such as depression, particularly in individuals with longer sleep durations. Our findings underscore the need for integrating sleep duration assessments into routine cardiovascular screenings.

This study has several limitations. Firstly, data on physical activity, dietary habits, and obstructive sleep apnea (OSA) were not collected, which could introduce bias in the observed associations. These unmeasured confounders are known to affect both sleep duration and cardiovascular outcomes. For instance, physical inactivity and poor diet are well-established risk factors for cardiovascular disease, and OSA is a common condition associated with both sleep duration and cardiovascular morbidity. The absence of these variables in the analysis may have led to an overestimation or underestimation of the true association between sleep duration and CAD. Additionally, residual confounding from these factors cannot be ruled out. Future studies should aim to include these critical variables and adjust for them in the analysis to provide a clearer understanding of the relationship between sleep duration and cardiovascular risk.

Although we used **Complete Case Analysis (CCA)** to handle missing data, this method may lead to a reduction in sample size, especially when there is substantial missing data, potentially affecting the representativeness of the sample. Thus, the results may be less generalizable. Future studies should consider employing more sophisticated missing data handling methods, such as **Multiple Imputation**, to mitigate potential biases from missing data. Clinicians and policymakers should emphasize sleep duration assessment in preventive care strategies, particularly in high-risk populations. Future research should adopt longitudinal designs and objective sleep measures to validate these findings and further elucidate causal mechanisms.

## 6 Conclusion

Our findings underscore the importance of maintaining a normal sleep duration (6–8 h/day) as a strategy to preventcoronary artery disease. Clinical and public health efforts should prioritize sleep health interventions, integrating routine sleep assessments into primary care for early detection and management.

## Data Availability

The raw data supporting the conclusions of this article will be made available by the authors, without undue reservation.
